# Toxic effects of brake wear particles on epithelial lung cells *in vitro*

**DOI:** 10.1186/1743-8977-6-30

**Published:** 2009-11-20

**Authors:** Michael Gasser, Michael Riediker, Loretta Mueller, Alain Perrenoud, Fabian Blank, Peter Gehr, Barbara Rothen-Rutishauser

**Affiliations:** 1 Institute for Anatomy, Division of Histology, University of Bern, Bern, Switzerland; 2 Institute for Work and Health, University of Lausanne and Geneva, Lausanne, Switzerland

## Abstract

**Background:**

Fine particulate matter originating from traffic correlates with increased morbidity and mortality. An important source of traffic particles is brake wear of cars which contributes up to 20% of the total traffic emissions. The aim of this study was to evaluate potential toxicological effects of human epithelial lung cells exposed to freshly generated brake wear particles.

**Results:**

An exposure box was mounted around a car's braking system. Lung cells cultured at the air-liquid interface were then exposed to particles emitted from two typical braking behaviours („full stop“ and „normal deceleration“). The particle size distribution as well as the brake emission components like metals and carbons was measured on-line, and the particles deposited on grids for transmission electron microscopy were counted. The tight junction arrangement was observed by laser scanning microscopy. Cellular responses were assessed by measurement of lactate dehydrogenase (cytotoxicity), by investigating the production of reactive oxidative species and the release of the pro-inflammatory mediator interleukin-8. The tight junction protein occludin density decreased significantly (p < 0.05) with increasing concentrations of metals on the particles (iron, copper and manganese, which were all strongly correlated with each other). Occludin was also negatively correlated with the intensity of reactive oxidative species. The concentrations of interleukin-8 were significantly correlated with increasing organic carbon concentrations. No correlation was observed between occludin and interleukin-8, nor between reactive oxidative species and interleukin-8.

**Conclusion:**

These findings suggest that the metals on brake wear particles damage tight junctions with a mechanism involving oxidative stress. Brake wear particles also increase pro-inflammatory responses. However, this might be due to another mechanism than via oxidative stress.

## Background

Ambient particulate matter (PM) was found to cause adverse health effects associated with increased pulmonary and cardiovascular mortality [1-4]. Traffic intensity is one of the most important determinants of ambient anthropogenic PM concentration, and people living in cities and near major traffic routes are particularly affected by high levels of PM pollution [1,5,6].

In Switzerland road traffic contributes about 30-40% of total PM10 emissions (PM smaller than 10 μm). For streets with a high traffic volume the contribution is about 45-65% [7]. These emissions contain PM from combustion but also abrasive wear from tyres and brakes [8]. Brake wear debris was estimated to contribute about 12.5-21% to the total PM traffic emissions in Germany [9].

Toxicity and health consequences of exposure to diesel exhaust particles (DEP) have been studied in great detail [10-14]. In contrast only few studies exist about specific health effects of brake wear PM. Riediker and colleagues [15] showed that a particle source with a brake wear signature was strongly linked to health effects. They found that PM2.5 originating from speed-changing traffic modulates the autonomic control of the heart rhythm, increases the frequency of premature supraventricular beats and elicits pro-inflammatory and pro-thrombotic responses in healthy young men. These health effects might be associated with the levels of the metals which are an important component of brake wear. It has been shown that elevated concentrations of metals occur inside cars [16]. Particularly copper was strongly increased and brake abrasion was proposed as likely source. A considerable fraction of freshly generated brake wear particles is smaller than 100 nanometres [17,18]. Such nanoscaled particles are believed to contribute importantly to the health effects caused by inhaled PM [19]. Beside the chemical composition and the size the toxicity of nanoparticles also depends on other properties [20,21] such as bulk material or surface charging [22]. Thus the potential toxicity of each class of combustion-derived nanoparticles has to be evaluated individually.

The goals of this study were to evaluate potential toxic effects of brake wear particles in lung cells and to further investigate the influence of particle size and -composition on these effects. Recently, we have established a system, which allows exposing cells directly to freshly emitted brake wear particles [23]. In the present study we have used this system to expose A549 epithelial cells, cultured at the air-liquid interface, to brake wear particles produced by different brake conditions, i.e. "normal deceleration" and "full stop". Components analysed were metals (iron, manganese and copper) as well as elemental and organic carbon were analysed on-line. Particles deposited on grids for transmission electron microscopy (TEM) have been counted to determine the number of particles which were deposited on the cell surface. The carbons were included because modern brakes contain a large amount of carbonaceous components such as polymeric binders. Post exposure analysis of the cells included the cell viability and the tight junction (TJ) arrangement, since we have recently shown that exposure to engineered nanoparticles leads to a disorganization of the TJ in A549 cells [24]. In addition, the formation of reactive oxygen species (ROS) and the release of the pro-inflammatory mediator interleukin-8 (IL-8) were investigated since it is known from earlier studies that exposure to PM leads to health effects via oxidative stress and inflammatory responses [10,13,25].

## Results

### Exposure experiments

A total of 14 experimental test runs were conducted (including the "no stop run"), resulting in 4-6 valid runs for each of the braking behaviours (Table 1). During all the experiments, temperature and humidity stayed constant within the limits required for cell culture experiments. The exposure conditions inside the exposure box are described in detail in the Material and Method part.

**Table 1:** The braking behaviours used in the present study.

„normal deceleration“	Acceleration in 3rd gear up to 2000 RPM (revolutions per minute). Two minutes of constant 2000 RPM were followed by a deceleration phase of ten seconds to 1750 RPM. The gas pedal position was unchanged during this deceleration phase to simulate the inertia of the car. 8 repetitions (8×) were performed in 16 minutes.
„full stop“	Acceleration in 3rd gear up to 3000 RPM. After two minutes of constant 3000 RPM, a „full stop“ was performed with the gas pedal position unchanged until the engine stalled. The engine then had to be restarted for the next cycle. 4 and 8 repetitions (4× and 8×) were performed in 8 and 16 minutes.

„no stop run“	Acceleration in 3^rd ^gear to 2'000 RPM with the speed remaining stable throughout the entire measurement cycle of 16 minutes.

### Particle characterisation

**Figure 1:**
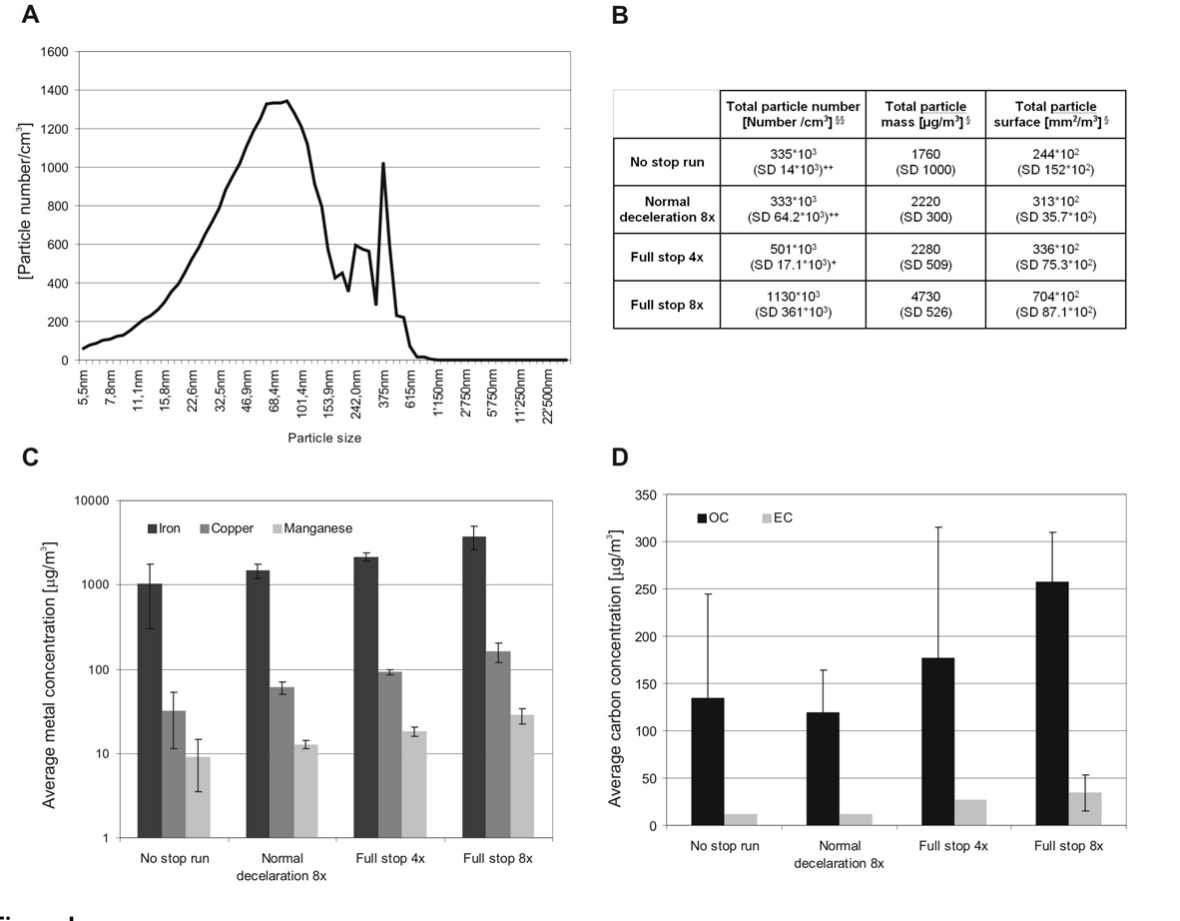
**Characterisation of brake wear particles**. A) shows a typical contribution of measured brake particles from a braking simulation with 8 repetitions, „normal deceleration“. B) shows the particle number, mass and surface concentrations in the air of different braking behaviours. Values represent the sum of all concentrations measured during one run ("sum under the curve"). Such a representation enables a better comparability among runs with different exposure times. SD represents the standard deviation. §p < 0.05, §§p < 0.01 represent a statistically significant influence of the fixed factors. + stands for p < 0.05, ++ for p < 0.01 in pairwise Bonferroni t-tests of designated braking conditions in comparison with the "full stop 8x". C) and D) represent identical illustrations with measured metals and carbon components. Displayed are the means and the standard deviations from 2 ("full stop 4x") or 4 (all the other conditions) experiments. Standard deviations for the elemental carbon could not be calculated for all bars because of values below the detection limit.

During both braking behaviours high quantities of nanoparticles (< 100 nm) and fine particles (100 nm - 2.5 μm) were produced (Fig. 1A and 1B). The typical distribution was twin or triple peaked (example shown in Fig. 1A) with a peak at about 100 nm and another at 300 to 400 nm. The total number of particles, particle mass and particle surface was measured in realtime during the exposures in the exposure box (Fig. 1B). The values represent the mean of the sum of all concentrations measured during one run. The particle number was significantly higher for "full stop 8x", i.e. 1130*10^3 ^number/cm^3 ^(SD 361*10^3^) than for all the other braking behaviours. For all 3 measured parameters the fixed factor braking behaviour showed a high significant influence. However, because of unequal variances no significant differences were found for the mass and the surface in pair wise comparisons.

Concentrations of the measured brake emission components in the air (metals and carbons) were increasing with more repetitions and for the more vigorous „full stop“. Iron was found at the highest concentrations (mean values in a range from 1020 μg/m^3 ^to 3770 μg/m^3^), followed by copper (33 μg/m^3 ^to 163 μg/m^3^), and manganese (9 μg/m^3 ^to 28 μg/m^3^) (Fig. 1C). Concentrations of organic carbon were considerably higher than elemental carbon concentrations (Fig. 1D). The mean values for elemental carbon ranged from 12 μg/m^3 ^to 35 μg/m^3^. Mean Values for organic carbon were between 120 μg/m^3 ^and 258 μg/m^3^.

**Figure 2:**
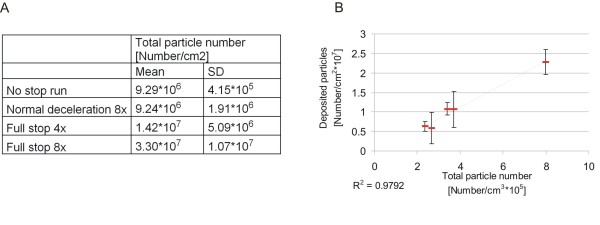
**Number of deposited particles and correlation of the number of deposited particles with total particle number**. A) Particle number/cm^2 ^was calculated by counting the individual particles deposited on a TEM grid. B) Total particle number (sum under the curve) was measured online during one exposure and correlated with the number of deposited particles.

Deposited particle numbers per cm^2 ^surface area were calculated by the simultaneous measurement of the total particle number in the box (sum under the curve) and the particles deposited in 5 additional runs. TEM grids were placed inside the box at the same place as the cells and were exposed to the particles during different braking behaviours. The number of particles from each exposure was analysed by TEM (Fig. 2A). We found for "no stop" 9.29 × 10^6 ^particles/cm^2 ^(SD 0.415*10^6^), for "normal deceleration" 9.24*10^6 ^particles/cm^2 ^(SD1.9*10^6^), for "full stop 4x" 14.2 *10^6 ^particles/cm^2 ^(SD 5.09*10^6^), and for "full stop 8x" 33*10^6 ^particles/cm^2 ^(SD 10.7*10^6^). The total number of deposited particles from the same experiments was then calculated then by using the plotted function. Figure 2B shows the correlation of the deposited particles per cm^2 ^surface area and the total number of particles in the air.

### Cytotoxicity and oxidative stress in A549 cells

**Figure 3:**
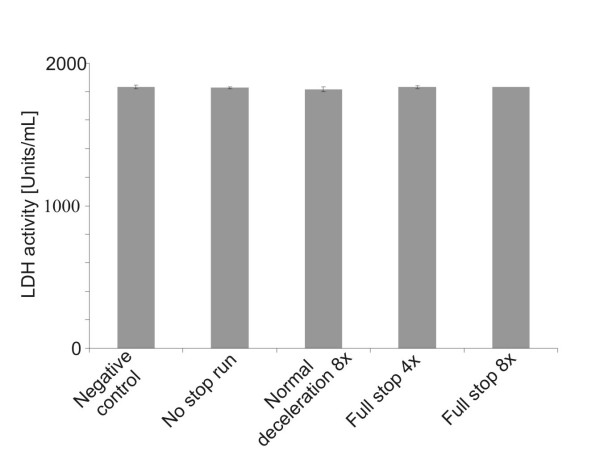
**Cytotoxicity in exposed A549 cells compared with control cultures**. LDH levels were determined as a marker of cell death and the values did not change significantly after exposure to brake wear particles. Each value represents the mean and standard deviations from 3 individual exposure experiments.

**Figure 4:**
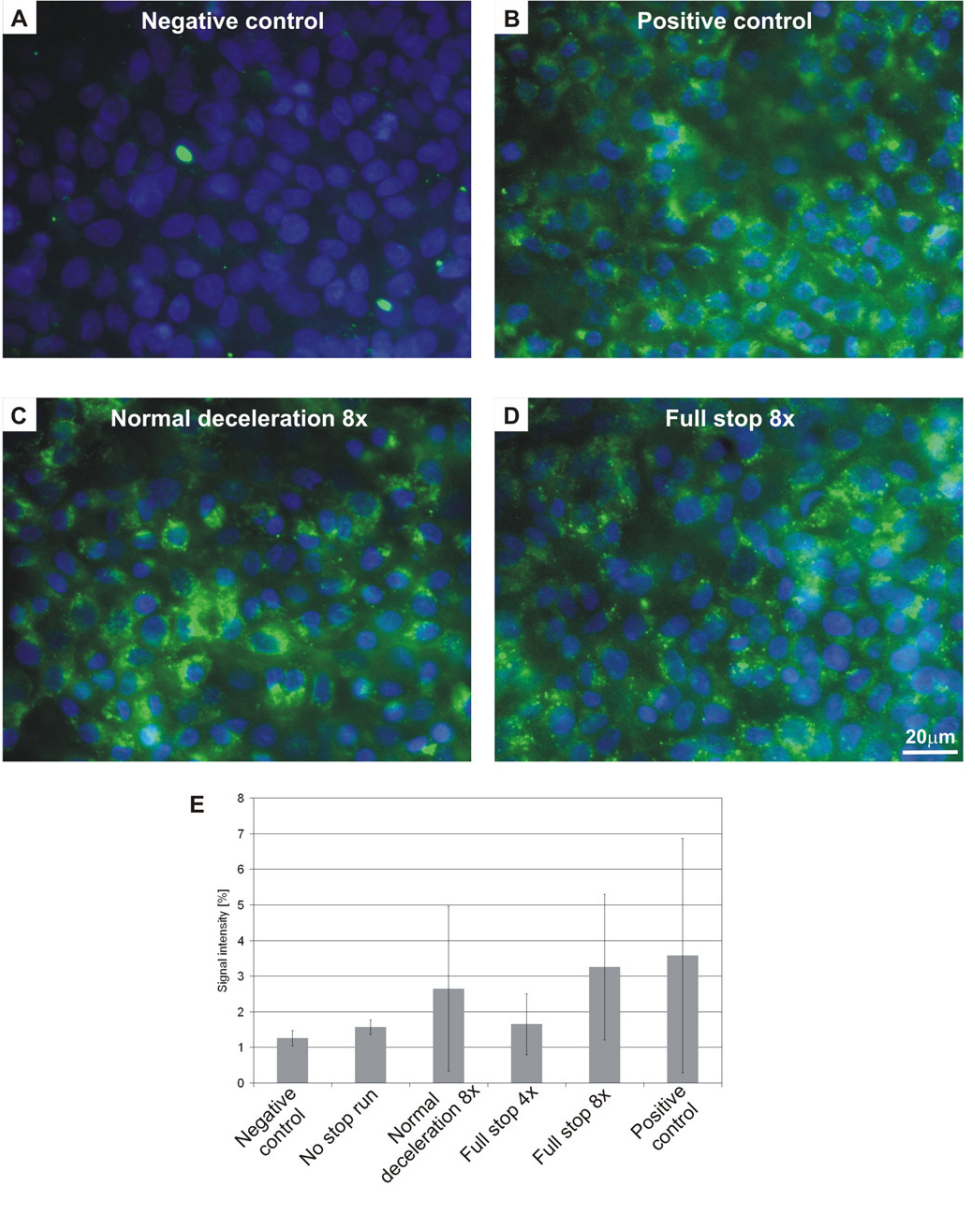
**Production of reactive oxygen species in exposed and non exposed cells**. Picture A) shows cells that were not exposed (negative). Picture B) stands for the positive control (treated with TBHP). Cells on pictures C) and D) were exposed to brake wear particles ("normal deceleration 8x" and "full stop 8x"). The Intensity of ROS from A549 cells exposed to brake particles was quantified with the software ITEM (E). Displayed are the means and the standard deviations from 2 ("full stop 4x") or 4 (all the other conditions) experiments.

The cytotoxicity was assessed by lactate dehydrogenase (LDH) measurements which showed no significant difference between the controls in the incubator or from the particle-exposed cells indicating that the cells were not damaged by the particle exposure in box (Fig. 3). The production of ROS as an indicator of oxidative stress was investigated in A549 cells using a fluorescence marker. In the negative controls, i.e. cells that were not exposed in the exposure box, only few cells were found with ROS signals (Fig. 4A); whereas most of the cells treated with tert-butyl hydroperoxide (TPHB; positive control) showed ROS signals (Fig. 4B). Cells exposed to brake PM showed more ROS signals than control cultures (Fig. 4C-D). Figure 4E shows values received from the fluorescence quantification of the pictures. More ROS signals for the "full stops" with more repetitions were measured, however, the differences were not statistically significant.

### Cell analysis

24 h after the particle exposure cells were fixed and stained for F-actin and visualized by laser scanning microscopy. In general the cell morphology was not affected by particle exposure when compared with non-exposed cells (data not shown).

**Figure 5:**
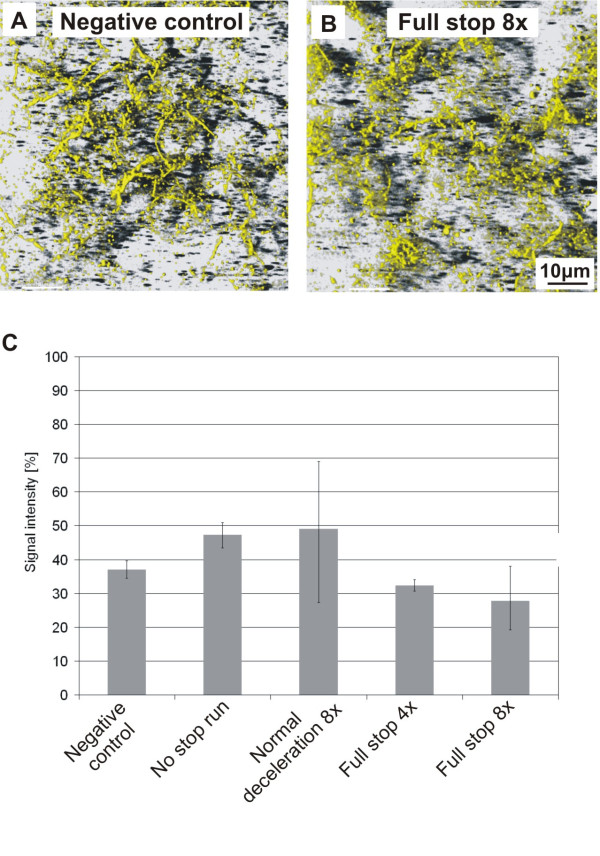
**Influence of brake wear particles on the tight junction protein occludin**. A) Non exposed cells. B) Cells exposed to a „full stop“ with 8 repetitions. The graph on the bottom shows the quantifications of occludin (B). Each value represents the median of the 25%- and the 75%-quantile from 2 ("full stop 4x" and "no stop run") or 4 (all the other conditions) experiments.

Analysis of the TJ protein occludin revealed some structural difference between control cells and exposed cells. In control cells occludin was localized at the cell borders and also some staining was seen in the cytoplasm (Fig. 5A), whereas in cells exposed to "full stop 8x" conditions the staining at the cell borders was reduced (Fig. 5B). A comparison of the medians from the quantified signals from pictures taken by conventional fluorescent microscopy (Fig. 5C) showed a tendency for less occludin in cells exposed to "full stops" particles.

### Release of the pro-inflammatory mediator IL-8

**Figure 6:**
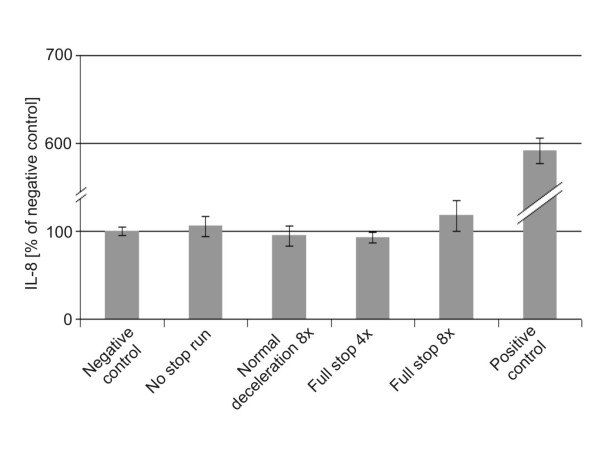
**Concentrations of IL-8 released from A549 cells exposed to different braking behaviours**. Means and standard deviations from 2 ("full stop 4x" and „no stop run“) or 4 (all the other conditions) experiments. A positive control was generated with TNF-α stimulation.

The concentrations of IL-8 in the cell culture medium, which was 2.5 mL (medium volume) in the lower chamber, were determined by ELISA. The final values are expressed as percentage of the negative control, which corresponded to 0.6 ng/mL. The increase of IL-8 was a factor 6 after exposure to tumour necrosis factor alpha (TNF-α) which was used as a positive control. There was a tendency to higher IL-8 levels for the "full stop 8x" braking behaviour compared to the negative control and the other braking behaviours (Fig. 6).

### Correlating factors

**Figure 7:**
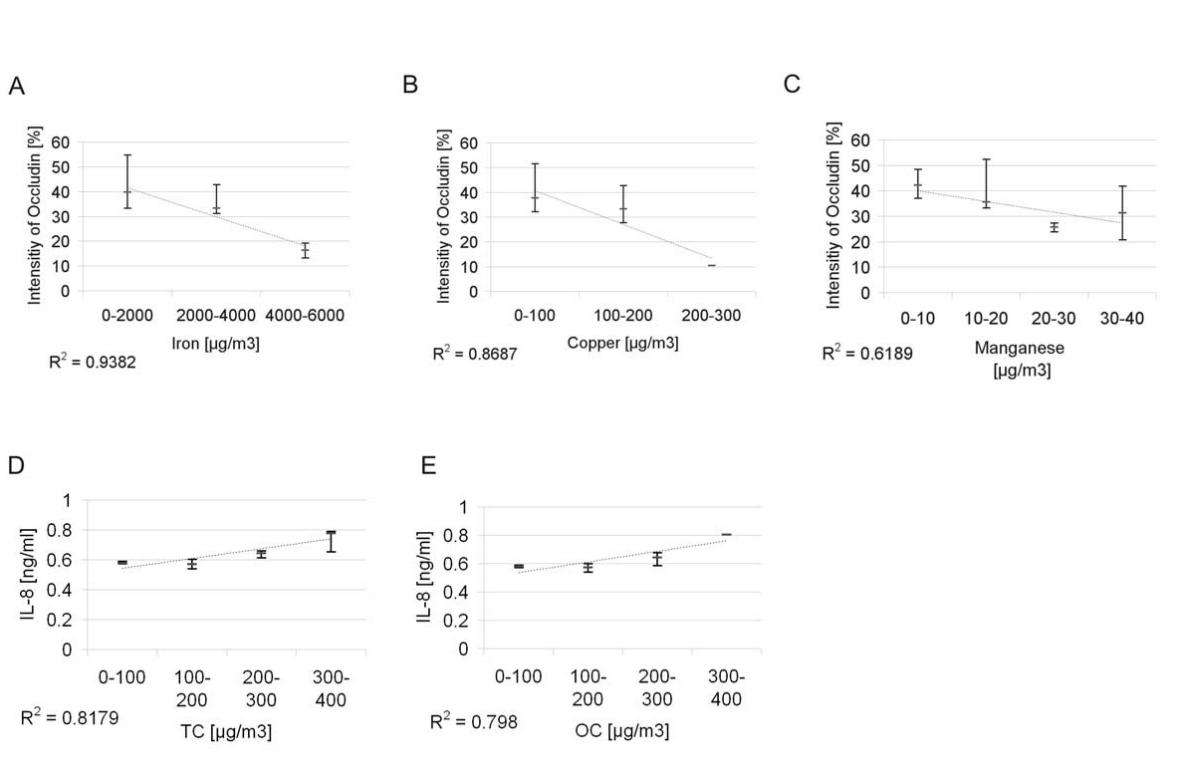
**Selection of correlating factors**. A-C shows the metals iron, copper and manganese correlating with occludin. D and E are the correlations of total carbon and organic carbon with measured IL-8 concentrations. The spotted line shows the linear correlation of the medians.

The quantified amount of occludin showed a statistically significant (p < 0.05) negative correlation (Fig. 7A-C and Table 2) with all 3 measured metals (copper, iron, manganese), which were also highly correlating (p < 0.005) among themselves (Table 3). Significant (p < 0.05) correlations were found between the measured carbons (total carbon and elemental carbon) and the IL-8 concentrations (Fig. 6D and 6E). Among the endpoints a highly significant (p < 0.005) negative correlation was found between ROS and occludin (Table 4).

**Table 2:** Spearman's rank correlations (2-sided significance) for fixed factors and endpoints. *p < 0.05.

	IL-8	Occludin	ROS
Iron	0.217	-0.678*	0.476

Copper	0.259	-0.615*	0.392

Manganese	0.224	-0.636*	0.434

TC	0.608*	0.014	0.028

OC	0.594*	0.049	0.000

EC	0.342	-0.577	0.523

Number	0.364	-0.301	0.196

Mass	0.231	-0.469	0.490

Surface	0.217	-0.385	0.385

**Table 3:** Spearman's rank correlations (2-sided significance) for fixed factors. *p < 0.05, **p < 0.01, ***p < 0.005.

	Iron	Copper	Manganese	TC	OC	EC	Number	Mass
Copper	0.974***							

Manganese	0.991***	0.960***						

TC	0.500	0.533	0.527					

OC	0.445	0.489	0.467	0.989***				

EC	0.883**	0.811*	0.955***	0.739	0.739			

Number	0.793***	0.798***	0.785***	0.593*	0.533	0.847*		

Mass	0.815***	0.780***	0.811***	0.451	0.401	0.847*	0.754***	

Surface	0.829***	0.776***	0.846***	0.549	0.478	0.811*	0.824***	0.952***

**Table 4:** Spearman's rank correlations (2-sided significance) for endpoints. ***p < 0.005.

	IL-8	Occludin
Occludin	0.196	
ROS	-0.226	-0.745***

## Discussion

Brake wear is an important source of traffic particles [9] and it is therefore important to assess its potential toxic risk on human lung cells. A system was established to measure the effects of brake wear particles directly emitted onto lung cells cultured at the air-liquid interface in a closed box [23]. Runs with different braking behaviours, i.e. „normal deceleration“ and „full stop“, and with several numbers of repetitions were conducted. We found that "full stop" produced the highest number and mass concentrations of fine particles and nanoparticles. Brake wear particles contain considerable amounts of iron, copper and organic carbon, which was negatively correlated with the quantified amount of occludin but not with IL-8. However, significant correlations were found between the measured carbon and the IL-8 concentrations.

There are only a few studies of airborne brake wear particles [18,26,27] and to our current knowledge we are the first to study the toxic effect of those particles in the present work. The interpretation of data has to taken into account possible discrepancies between different studies as well as of different brakes. In the present study we have included two important controls which allow interpreting the observed effects. First, for each exposure we have included a negative control, which were cells incubated for 24 h at the air-liquid interface in the incubator. Second, we have exposed cells for the same duration in the exposure box during the "no stop run" braking behaviour. All following tests were done exactly at the same time and using the same assays as for the exposed cells.

To assess the potentially adverse toxic potential of brake wear particles, we have exposed them onto A549 cells which were placed within the exposure box. The A549 cell line originates from human lung carcinoma [28] and is routinely used as an *in vitro *model of pulmonary cuboidal epithelium to study the interaction of environmental particles [29] with the cells. It has also been shown that A549 cells, when cultured at the air-liquid interface, express TJ and produce surfactant [30,31], which does reflect the physiological condition of these lung epithelial cells when they are exposed to air. A549 cells cultured at the air-liquid interface are routinely used to assess the toxic effects of volatile organic compounds [32] or particles, as for instance carbon nanoparticles [33] or cerium dioxide nanoparticles [24]. The use of the A549 cells as a system to assess effects upon particle exposure as well as all cellular assays have been established in earlier studies [24,30].

The total particle number during the exposure in the box was significant higher during the "full stop" decelerations than during "normal deceleration" and "no stop run" conditions. For "full stop 8x" a total particle number of 1130*10^3^/cm^3 ^(SD 361*10^3^) with a peak at about 100 nm and another at 300 to 400 nm was measured. A recently published paper [34] indicated an elevated mortality risk from short-term exposure to combustion-derived nanoparticles. The monitoring site was representative for the air quality in the city Erfurt and the authors have found that the interquartile range increase in a 15-day cumulative mean of nanoparticles of 7649/cm^3 ^was associated with an increased mortality. Our measured total particle number at "full stop 8x" was much higher, but also in a defined volume (the exposure box), however, future activities should go in the direction to produce brakes with no abrasions during braking.

The "no stop runs" showed a relatively high PM concentration and were in the same range as the "normal deceleration 8x" runs. A possible explanation might be that particles adhered to the braking system and were released when the brake disc was turning. This finding is supported by the study from Gehrig et al. [35] who found brake wear components in PM sampled along highway segments where few braking events are expected. The fact that the particle concentration between "no stop run" and "normal deceleration 8x" were in the same range can explain that we did not found any difference in cellular responses for all assays we have looked at.

The deposition of particles on the cell surface has been estimated by counting particles on TEM grids. We have found the following depositions: for "no stop" 9.29 × 10^6 ^particles/cm^2 ^(SD 0.415*10^6^) up to 33*10^6 ^particles/cm^2 ^(SD 10.7*10^6^) for "full stop 8x". This is the first published study describing the deposition of brake wear particles onto cells. However, in a study published by Holder and colleagues [36] diesel exhaust particles were exposed to the air-liquid interface of cell cultures. The deposition of particles was assessed in a similar way as we have done it and a number of 23*10^6 ^particles/cm^2 ^was found which is in agreement with the numbers we have calculated for "full stop 4x" and "full stop 8x". However, the responses can not be compared because of the different used cells and the different particles used in the experiments of the two different laboratories.

One aim of the present study was to assess the localisation and intensity of the TJ protein occludin in the A549 monolayer because the same cell culture system was used in a similar study, which demonstrated a decrease of the fluorescent signal of occludin and a decrease in permeability of the epithelial monolayer after exposure to cerium oxide particles in a dose-dependent manner [24]. The mean fluorescence intensity of occludin immunostainings increased for the "normal deceleration", whereas for the other braking behaviours used in the present studies the signal decreased. There might be an effect from higher particle concentrations. TJ proteins are very dynamic and change their location when the permeability of the epithelial monolayer is changing [37,38]. Yshii and colleagues [39] exposed mice as well as *in vitro *cultured alveolar epithelial cells to PM10 and demonstrated a decrease in the total abundance of occludin. They also observed that connections of occludin with the protein zonula occludens-1 were altered. The latter is important for the connection of the tight junctions with the cytoskeleton. If these mechanisms are affected the same way from brake wear particles this would mean that the tightness of the epithelial barrier decreased. In our case at least one of the measured metals could play a key role since iron, copper and manganese show a significant influence on the measured occludin intensity. However it is difficult to assess the relevance of each single metal in affecting occludin expression (all of them correlate with each other) and to our knowledge there is no mechanism described about the influence of these metals on occludin.

Cell viability was not impaired, however, we observed oxidative stress in cells, which was induced after exposure to particle emissions mainly from „full stop“ braking with more repetitions. In addition, we found a tendency to higher IL-8 levels with increasing number of braking events and braking strength. Most of the data about the mechanism of the adverse health effect of PM suggest that oxidative stress plays a key role [10]. Further associations between oxidative stress and inflammation responses are described in the literature [13,25,40] and inflammation responses are associated again with adverse health effects [4,5,41]. From our metal analysis we have seen that iron was produced in the highest concentrations. Aust et al. [42] reported that iron from coal fly ash acts as a catalyst for redox reactions leading to oxidative stress in vitro. They also found that more iron was released from the smaller particles (PM1, PM2.5) than from larger ones (PM10). Furthermore, correlations to inflammatory responses were made. In their study IL-8 concentrations were higher when cells were exposed to small particles. IL-8 is an important chemokine that is released in the pro-inflammation process by epithelial cells. Analysis showed that with more repetitions and therefore higher particle concentrations, more IL-8 was produced. It has been shown that carbonaceous nanoparticles induce IL-8 gene transcription in normal human bronchial epithelial cells [43]. We can see an analogy to the significant correlation we observed between the total measured carbon and the measured IL-8 concentration. Therefore we might assume that high concentrations of carbon in combination with the small size of the particles can increase IL-8 concentrations. In another study similar concentrations of IL-8 (0.4 ng/ml) were detected when A549 cells were exposed to DEP PM1 [0.1 mg/ml] using the same incubation time we used [44]. We have measured 0.55 ng/mL IL-8 for "full stop 4x" and 0.7 ng/mL IL-8 for "full stop 8x". A direct comparison, however, is difficult since our study was done at the air-liquid interface and the latter one in suspension.

## Conclusion

The direct combination of lung cell culture exposure to brake wear particles offers a reliable approach to investigate the cellular effects of directly emitted particles. For all measured parameters the „full stop“ with the most repetitions provoked the most adverse effects, i.e. increase in oxidative stress and pro-inflammatory response in lung cells. The exposure to brake wear particles causes the formation of ROS for both braking behaviours. For particles emitted by „full stop“-behaviours there is a potential to affect the TJ arrangement, as well as an increased IL-8 release by the cells.

We found a negative correlation between iron, copper and organic carbon contents in the brake wear particles with the quantified amount of occludin but not with IL-8. However, significant correlations were found between the measured carbons and the IL-8 concentrations. Therefore we assume that brake wear particles induce oxidative stress and pro-inflammatory reactions but that different pathways activated by the various components are involved.

For future studies effects will be appraised and different brake materials need to be compared concerning potential health effects. Like for exhaust particles, efforts to diminish brake particle emissions will lead to an improved ambient air quality and so could provide a better protection of human health.

## Methods

### Cell cultures

The A549 cells (LGC Promochem, Molsheim, France) were cultured as described by Blank et al., 2006 [30]. Briefly, the cells (passage number 15) were maintained in RPMI 1640 medium (w/25 mM Hepes; LabForce AG, Nunningen, Switzerland) supplemented with 1% L-Glutamine (LabForce AG), 1%penicillin/streptomycin (Gibco BRL, Invitrogen AG, Basel, Switzerland), and 10% fetal calf serum (PAA Laboratories, Lucerna-Chem AG, Lucerne, Switzerland). Cells were seeded at a density of 0.5 × 10^6 ^cells/ml on BD Falcon cell culture inserts (surface area of 4.2 cm^2^, pores with 3.0 μm in diameter, high pore density PET membranes for 6 well plates; BD Biosciences, Basel, Switzerland). Inserts were placed in BD Falcon tissue culture plates (6 well plates; BD Biosciences) with 2 ml medium in the upper and 3 ml in the lower chamber. Medium was changed twice weekly. Cells were grown on inserts submersed in medium for 7 d to grow to confluence. Then the medium was removed from the upper chamber while the medium in the lower chamber remained to supply the cells through the membrane. However, its volume was reduced to 2.5 ml to reduce the liquid pressure from the lower chamber. The cells were then exposed to air for 1 d before being used for the particle exposure experiments.

### Exposure system

The system used here is described in Riediker et al. 2008 [23] and in Perrenoud et al. (Perrenoud A, Gasser M, Rothen-Rutishauser B, Gehr P, Riediker M: Characterization of nanoparticles resulting from different braking behaviours, submitted). Briefly, a Renault Laguna 2.0 was installed on a lift in a garage and one front wheel was removed. Subsequently an exposure box was installed around the axle and the brake with all sides closed with aluminium foil (Korff, Oberbipp, Switzerland). Before the cells were exposed the exposure box was closed and filtered sterile air (Filter: HEPA-CAP 36, VWR International, Dietikon, Switzerland) pumped into the interior. Inside the exposure box a self-made air-cooling-system and a heated water bath were installed to provide adequate conditions for the cells (in-well temperature 37°C, relative air humidity between 40% and 80%). Through a flap door on one side the 6 well plates with the cells were placed into the exposure box. A glove beside that flap door and a glass window on the upper side of the exposure box enable manipulations within the exposure box. After the 6 well plates were placed on the water bath, the exposure box remained closed to flush it with particle-free air. 10 min later the covering of the 6 well plates was removed and the braking procedure started. Two braking behaviours were tested (simplified from Sanders et al. [26]). The "normal deceleration" simulates typical urban driving behaviour and the „full stop“ stands for vigorous braking (Table 1). As a control a rotation without deceleration was performed (Table 1).

With the measurements of the particle number, mass and surface and with several chemical analyses important properties were characterised. After the braking procedure the cell cultures remained uncovered in the exposure box for 10 more min. They were then covered, taken out of the exposure box and placed back in the incubator for additional 24 h. A negative control was performed by incubating cells at the air-liquid interface for 24 h in the incubator.

During the exposure, the following parameters were measured: humidity, temperature, relative polycyclic aromatic hydrocarbon (PAH) concentrations, and particle size, surface and mass. In addition, PM2.5 was collected on filters and analysed for carbon and metal content. Organic resins are frequently used as binders in brake pads. During the braking procedure a part of the organic carbon may be pyrolized and thereby converted to elemental carbon. To assess these two fractions filters were analysed for organic and elemental carbon compounds.

### Transmission electron microscopy (TEM): quantification of particles

Parallel to the exposure of cell cultures, copper TEM grids were placed in the 6-well plates inside the exposure chamber and exposed to the brake wear particles in 5 additional runs. This allowed to determine the number of particles which were deposited on the cell surface. Therefore the grids where analyzed in a systematic way using the 11.500× extension of a Philips 300 TEM at 60 kV (FEI Company Philips Electron Optics, Zurich, Switzerland). One end of the grid hole was searched, a picture was taken and then the next picture was taken ten half turns in the opposite direction, away from the end. This procedure was repeated till the other end of the grid was reached. The particles were counted manually. The level of blank grids (equally treated but unexposed) was taken as background and subtracted from the other values.

### Cytotoxicity

In order to determine cell death the release of lactate dehydrogenase (LDH) from necrotic cells was measured. The supernatant in the lower well was collected after particle exposure followed by the 24 h incubation time and analysed by using a colorimetric LDH cytotoxicity detection kitPLUS (Roche Applied Science, Mannheim, Germany) according to the supplier's manual. Each supernatant was measured in duplicates.

### ELISA

24 h after particle incubation, medium from A549 cultures in the lower chamber was collected and stored at -80°C. After centrifugation, Interleukin-8 (IL-8) was quantified by a commercially available DuoSet ELISA Development kit (R&D Systems, Catalogue Number: DY 208, Oxon, UK) according to the manufacturer's recommendations. The only modification was that after 20 min in darkness, the colour development was stopped with 2NH_2_SO_4 _and the plate was placed on a shaker for 10 min. Then the absorbance was read at 450 nm using an ELISA reader (SpectraMax 340 PC or Benchmark Plus Microplate Spectrophotometer (BioRad, Hempel Hempstead, UK)). Exposure to TNF-α (Sigma-Aldrich, Switzerland) (10 ng/mL) was performed as a positive control for IL-8 induction.

### Detection of reactive oxygen species

A ROS detection kit was used to detect oxidative stress (Image-iT™ LIVE Green Reactive Oxygen Species Detection Kit, Molecular Probes, Invitrogen AG, Basel, Switzerland). Briefly, intracellular nonspecific ROS were marked with a carboxy-H2DCFDA solution and the cell nuclei were stained with Hoechst 33342. Afterwards, cells were washed with Hank's balanced salt solution (HBSS;Gibco BRL, Invitrogen AG, Basel, Switzerland) and fixed for 15 minutes at room temperature in 3% paraformaldehyde in PBS. After washing the cells were embedded in Mowiol 4-88 (Calbiochem, VWR International AG). The positive control was prepared with tert-butyl hydroperoxide (TBHP) according to the manufacturer's protocol.

### Cell fixation and labelling

Cells were fixed and labelled as previously described [45]. Antibodies were diluted in PBS as follows: rabbit anti-human occludin 1:20 (71-1500, Zymed, P. H. Stehelin & Cie AG, Switzerland), goat anti-rabbit cyanine-5 1:50 (AP187S, Chemicon, VWR International AG, Life Sciences).

### Fluorescence microscopy

The samples were imaged using a Leitz DMDR fluorescence microscope (Leica Microsystems AG, Glattbrugg, Switzerland) with an Olympus Digital Camera (C-3000 Zoom, with the objective C3030-ADU). The image grabbing and the processing were done with the analysis software (Olympus Soft Imaging Solutions GmbH, Munster, Germany).

### Laser scanning microscopy and image restoration

A Zeiss LSM 510 Meta with an inverted Zeiss microscope (Axiovert 200 M, Laser: HeNe 633 nm) was used. Image processing and visualization were performed using IMARIS, a 3D multi-channel image processing software for confocal microscopic images (Bitplane AG, Zurich, Switzerland).

### Quantification of the fluorescent signals

Pictures taken with the Leitz DMDR fluorescence microscope were processed with the iTEM 5.0 software (Olympus Soft Imaging Solutions GmbH, Munster, Germany). The total intensity signal of each picture was quantified as follows: for the quantification of occludin an intensity threshold was fixed arbitrarily, and then the percentage of the total area which exceeded that value was measured for each picture. For each run, the mean intensity was then recorded. Quantification of ROS was conducted identically to the occludin detection. Additionally to the intensity measurement, we also quantified the ROS levels from each braking run by rating the ROS intensity. From each run several pictures were taken and each of them was categorised in "no ROS signal", "weak ROS signal", "medium ROS signal" and "very clear ROS signal". The rating procedure was conducted twice by two different persons. Each braking run got then a score by calculating the mean of the ratings of its pictures.

### Statistics

Statistical analysis was performed with Microsoft^® ^Excel 2000 for Windows, with SPSS 15.0 and with SigmaStat 3.5. To test the influence of the fixed factors exposition and type of braking on the measured variables, a Kruskal-Wallis H-Test was conducted. In case of a significant influence of a fixed factor, a Tamhane 2 Post-Hoc-Test or a Bonferroni t-test (for equal variances) was carried out to compare variables among each other. A Spearman's rank correlation coefficient was calculated to test for correlations between the different factors.

## Abbreviations

DEP: Diesel exhaust particles; IL-8: Interleukin-8; LDH: Lactate dehydrogenase; PAH: Polycyclic aromatic hydrocarbon; PM: Particulate matter; PM10: Particulate matter with an aerodynamic diameter smaller than 10 micrometers; PM2.5: Particulate matter with an aerodynamic diameter equal smaller than 2.5 micrometers; PM1: Particulate matter with an aerodynamic diameter smaller than 1 micrometer; ROS: Reactive oxygen species; TBHP: Tert-butyl hydroperoxide; TEM: Transmission electron microscopy; TJ: Tight junctions; TNF-α: Tumour necrosis factor alpha

## Competing interests

The authors declare that they have no competing interests.

## Authors' contributions

MG, AP, MR, PG and BRR carried out the design of the study. MG and AP collected, analysed and interpreted the data. MG drafted the manuscript. MR conceived and led the project and organized the funding. FB and LM were involved in the analysis and interpretation of data, as well as drafting the manuscript. BRR was the project leader for the cell testing and all related analyses. MR and PG have intellectually accompanied the cell experimental work; they were involved in revising the manuscript critically for important intellectual content and gave final approval of the version to be published.
